# President’s Address

**Published:** 1896-12

**Authors:** 


### PROCEEDINGS.

[*Continued from Page* 567.]

JUNE 11, 1896—MORNING SESSION.

The meeting was called to order by President House.

The minutes of the previous session were read and approved.

A paper entitled "What Should the Dentist do to Save the Teeth," by N. S. Hoff, D. D. S., was presented (See August No. of REGISTER, page 379).

The discussion was opened by Dr. Taft, as follows:

GENTLEMEN OF THE SOCIETY: The suggestion was made to me a few days ago to discuss this paper, and, especially, to discuss one of the features given in the first part of the paper, "What can be done by and for the expectant mother during the time of gestation and lactation, for improving the condition of her offspring?" This is a question that has been somewhat discussed, and upon which various opinions have been given; of course, practice has been regulated somewhat by these opinions, because, as a man believes, he will practice. There has been a diversity of opinion, not only among dentists, on this question, but with physicians as well; many dentists and many physicians believe that little or nothing can be done in the way of special treatment with this object in view; others, probably from experience or theories formed, maintain that much may be done. I believe there is a rational ground in reference to this matter that is tenable, and ought to be followed up, perhaps, more closely than it is. It is a question to which all should give attention, the solution of which—if there is as much in it as many of us think—would result in immense benefit to those who are to be mothers, to themselves, and to their offspring as well. I have no doubt that in a great many instances there is deficiency in the functions of maternity during gestation, a deficiency of some sort, a deficiency either of function or of supply that results in a marked defect in the teeth—and this, perhaps, interests dentists more than others—and in other tissues of the body as well as the teeth, and especially the bony structures of the body. This may occur from defective function, irrespective of supply, or the function may be good, or comparatively good, and yet a defective supply. In either case there will be imperfect tissue and structure formed. Now, it is true that the mother may be, in some respect or another, unable to take and properly prepare, in Nature's laboratory, the material necessary for her own support as well as for the support of her offspring—the child that is being formed. She may have one affection or another—a deficiency or shortness in function that renders it impossible for her organization to take the material presented and so utilize it; produce such changes upon it that it will be in a suitable state for the intended purpose. There is, doubtless, great variety in this respect; some may have a slight failure, others more marked—others more marked still—until, in many instances, a very great deficiency is present in the matter of digestion, assimilation and up-building of the material; there can be no question about that. We find mothers, many a time, in a condition where it is quite evident to the close observer and student of this question, that her offspring will necessarily be defective, puny and ill-developed in regard to bony structures, and probably other structures as well.

Now, what can be done for remedying this condition? The remedy here looks to general treatment to build up, to strengthen the system; to aid the digestive, nutritive, and assimilating functions; strengthen that as thoroughly as possible. Well, what does this mean—who is to do it; who is to recognize these conditions? Shall it be turned wholly over to the physician, and the dentist venture to make no suggestion to anybody about it? That would not seem to be a very rational course. The dentist necessarily deals with mothers during the time of gestation and lactation, and is it reasonable that he should know nothing about these things? If we conclude that this is the business of the physician, the mother ought to know from her physician what ought to be done in these respects—it is competent; it is not only proper, but it is a matter of right and duty that the dentist should know about these things—make them a matter of study, so that he may be able to recognize the ill-condition that is present and be able to suggest, at least, to the physician what ought to be done.

Then, there is an embarrassment here sometimes; the dentist will go to the physician and give his views, make his explanation in regard to these things, and describe what he, from observation, experience and study has been thoroughly convinced is true, and has a substantial basis for; he presents this to the physician many times, and he simply "pooh-poohs," and says: "You can do nothing for them; we can do nothing more than see that her environment and food are in good condition." But, happily, there are many physicians who take different views of this question. Knowledge is increasing; ability to understand these things is enlarging, and we find very many physicians who will take great interest in any suggestion made by the dentist.

Then, in regard to the matter of the nutritive process. The supply of material for the hungry organism is a great question; one that every dentist ought to study; one, the emergency of which, he ought to be able meet. Then, less frequently will be found, perhaps, a deficiency of supply in the food; that is not of very frequent occurrence, the great difficulties here lie along the line I have suggested—a want of ability on the part of the mother to properly prepare, assimilate and build into the growing tissues, or the tissues to be sustained, the material necessary for their best make-up. That I take to be the great difficulty, far more extensive than a deficiency in the taking of the material. I presume that in a large proportion of the food of mothers there is usually enough of the material for bone-supply to feed the growing bones and to supply the nutrition of the mother, but somehow or other there is a deficiency in the make-up of the teeth in this respect. We do find teeth of children many a time that are soft and defective from the very beginning, from want of proper supply; the mother is likely to suffer as well as the child. Who does not know that the teeth of the mother during gestation and lactation are much more sensitive than formerly? They are more liable to decay, so far as the dentine is concerned, at least; they are softer, and a change is perceptible at such times in many instances.

The increased tendency to, and occurrence of, decay of the teeth, during these periods, is not due wholly to the changed condition of secretions of the mouth, although there is matter there for investigation and for study, but to the defective condition of the digestive function; and it is due also to the deficient supply of material to be built into the structure at the time of its make-up. It is important, then, that this subject should have our special attention—it should not receive the "go-by" of the dentists.

Note a remark upon the third page of the paper: "Dentists, from the very nature of their work and practice, can not be expected to make accurate diagnoses nor to intelligently prescribe the proper quantity or quality of food for the great variety of physical conditions of the women of our day, but it is my opinion that we should not altogether ignore this matter, and that we should make ourselves as familiar with its general bearing as possible." Make ourselves familiar with the general condition and then follow the dictates of knowledge attained in that way, and we will have accomplished all that will be required. But he states, in the first of this paragraph, "it is not to be expected that the dentist will be able to make accurate diagnoses." Why not? Why isn't he capable of understanding these things? Simply because his attention has not been specially directed to these things, as has that of the physician. It is true that many dentists do not have a very full appreciation of this question; some do understand it quite well. The dentist can render valuable aid to the physician in this matter, and he can determine results with a considerable degree of accuracy. If he can gain knowledge that will be helpful to the physician, why not give it first-handed to his patients? You say, "The dentist can not do that." That is true of some, but many dentists can; those of large experience, extended observation and study, and familiarity with the subject, it is just as competent for him to suggest a certain course of living or a certain course of treatment, perhaps, as for the physician, so far as it pertains to the health of the teeth of the mother and the child. Many say, "Well, this is a delicate subject; I can not approach my patients on this subject, they will not take it kindly." That is a mistake; every intelligent mother, or prospective mother (with one who fully understands the subject; with one who can give her information that will result in her good and the benefit of her offspring) will take the most intense interest in the questions, and will receive everything looking in that direction with the greatest pleasure. All persons are not alike competent to present this subject; of course a younger dentist would ordinarily hesitate to present this subject to many of his patients, possibly to any of them. He says, "It is too delicate a question for me to deal with." The probability is that in the great majority of such cases that delicacy arises from the fact that he recognizes his own incompetency in reference to the matter. Usually, the man who knows the ground upon which he stands, and knows that he is competent to give advice to the patients that will be of great benefit to them, will find no difficulty in finding language or in finding means of conveying his information to those persons, and very rarely, indeed, will it ever be received other than with the greatest approval and delight. Every dentist who has been in practice for a long time, and who has given attention to this subject, will verify what I say. I know that a great many patients think that about all a dentist is expected to do is to fill decayed teeth and insert artificial teeth, to extract diseased teeth, etc., but fortunately, those entertaining such views are becoming less and less all the while, and it is so because the dentists are becoming more and more intelligent themselves, and are able to bring their patients up in this matter. Then let us give attention to this matter. It is not often that the dentist needs to refuse to see the physician, but will make his assistance serviceable so far as possible, and the younger members of the profession, especially, should do this. The older members will at once recognize the difficulties; recognize the things that ought to be done, and if they have the proper spirit they will do what they can. If there is any particular in which they think matters can be improved by consultation with others (either a dentist or physician), let that consultation be had; let the subject be discussed, and if it is better that the physician of the patient should have charge, with the suggestions of the dentist, let that course be pursued. How often is it we find mothers suffering greatly in various ways, and, it may be, with the parts adjacent to the teeth, resulting because of the conditions brought about by the maternal functions! How often are the teeth of the mother decayed and exceedingly sensitive; she can not masticate her food; the food is bolted, perhaps, or the food that is best for her is not taken. Then let us, as dentists, do whatever we can in this direction to be helpful to those who are suffering: this matter of improvement of the race is a great question, it has many branches, and this is not one of the least of those branches.

"It is high time that more notice was given to the eruption of the teeth in the regular order and harmonious position." Not only can the dentist render aid to the physician in this matter, as suggested, but he should, at least, be able to determine the comparative value of the different treatments, different methods of treatment in the results secured, and be able to fairly compare their value. In reference to irregularity of the teeth—that is another line of practice that ought to receive special attention. It is important that the teeth and all bony structures of the growing child be well developed, and have the proper supply of nutritive material in order that the teeth may be not only structurally good, but that they may have the proper arrangement in the arch. There is, as the paper says, usually no difficulty in this respect with the temporary teeth. There is, however, oftentimes very defective temporary teeth as the result of rickets and kindred affections. This, however, does not make the temporary teeth usually irregular in position or standing, but oftentimes teeth are missing because of this, and occasionally out of proper position. I remember a case of a little boy whose temporary teeth were scattered as though they had been sown broadcast. It is important to give attention to the period of the growth of the temporary teeth for the sake of the permanent teeth, because if the temporary teeth are defective in any way there is the greater probability that the permanent teeth will be ill-arranged. It is, perhaps, of less consequence to the temporary teeth because they are short-lived, but it is of great importance to the second or permanent set. This, in the apprehension of some, might lessen the regard or esteem which they would have for the temporary teeth. Magnify that as much as you can—let the thought be entertained that the temporary teeth are important, and, in a variety of ways, they are necessary in mastication. They serve a valuable purpose in promoting the growth and proper development of the parts in which they stand for the sake of the permanent teeth. In these respects the temporary teeth should not be lightly esteemed. This want of appreciation of the value of temporary teeth is expressed in this way by mother and children: "Oh, that is a temporary tooth," and so they give it little or no attention. Even the dentist, too often, yields to that kind of suggestion rather than correct it, when, by the treatment pursued under such circumstances, many times, the most serious consequences occur to the permanent teeth. We can hardly make a comparison as to the relative value of the permanent and temporary teeth. Of course, the permanent teeth serve much longer than the first, but the first teeth are important, and we ought not to cast a shadow of doubt over our opinion as to their importance.

The paper contains the suggestion that the free administration of bone-making material, and especially the calcareous, is likely to promote or induce an abnormal growth of the bony tissue throughout the body and so make difficult parturition. I apprehend that there need be no anxiety in regard to this question. Various authors make the statement that only so much of this material administered to the mother for this purpose as is needed will be taken, digested and built into the structure; that any excess passes off as any other excess of food will. We know that a larger amount of food may taken than is needed, altogether more than ought to be, but it does not follow that it will all be assimilated, only the needed amount to satisfy the hunger of the parts, or to satisfy the demand for the support or up-building of the tissues will be utilized; any excess will be rejected. So, there need be no fear entertained in regard to this matter in the administration of those preparations, and, besides, we should remember that the growth of the body, the form it takes, the size of it, etc., is according to a pre-arranged plan.

It is fully recognized in the medical profession that the use of bone-phosphates, or bone-making material, is very beneficial in certain conditions and in certain diseases, and so they are largely administered. Who does not know of the great rush there was some years ago for hypophosphites for consumption, and for various other affections? More than forty years ago experiments were made in the administration of lime salts to children, and to mothers for defective teeth, and it was observed then that very great benefit was derived from the administration of even this material in the crude form in which it was then used. From that time to this there have been many preparations made for the purpose of rendering this material more easily used by patients. I never saw a more pronounced result in the administration of lime phosphates than was observed in a treatment by the late Dr. Watt, forty-five years ago, in the case of a mother who had lost nearly all her teeth. Her first two children had very defective teeth; the teeth erupted with difficulty; when they came through they decayed rapidly, the temporaries and the permanent as well. During the time of gestation of the third child bone-phosphate was freely administered to the mother in a crude form, of course, because the good preparations we have now were not then extant. The third child erupted its temporary teeth promptly; they were good. I watched those teeth until they were shed; scarcely any of them decayed; the permanent teeth came through without difficulty and in regular form. Another child after that came, and the mother was subjected to the same treatment during the time of gestation and lactation. The fourth child had as good set of teeth as the third. The first two children lost their teeth, many of them at least, before the time of maturity; they began early to decay. This experience was sufficient to warrant further procedure of this kind, and from that day to this that kind of treatment, in the hands of a few at least, has been followed up with as good results as any we obtain in ordinary practice, not always, of course, with the same results, but in ordinary persons of good constitution, and where they observe right hygienic living and have good nutrition there will be benefit derived.

In the discussion of Dr. Hoff's paper Dr. Cleland advocated the use of coarse foods, such as oat-meal and milk (not cream), and bread and milk for children, also plenty of out-door exercise and fresh air.

